# Pancreaticoduodenectomy performed for a patient with prepancreatic postduodenal portal vein: a case report and literature review

**DOI:** 10.3389/fmed.2023.1180759

**Published:** 2023-08-16

**Authors:** Bingjun Tang, Sijia Li, Pengfei Wang, Jiming Ma, Fei Yu, Jun Shi, Xuedong Wang

**Affiliations:** ^1^Hepatopancreatobiliary Center, School of Clinical Medicine, Beijing Tsinghua Changgung Hospital, Tsinghua University, Beijing, China; ^2^Department of Gastrointestinal Surgery, School of Clinical Medicine, Beijing Tsinghua Changgung Hospital, Tsinghua University, Beijing, China

**Keywords:** prepancreatic postduodenal portal vein, pancreaticoduodenectomy, case report, portal vein variant, pancreatic surgery

## Abstract

**Introduction:**

Prepancreatic postduodenal portal vein (PPPV) is a rare congenital variation, with only 17 cases reported in the literature and five of them undergoing pancreaticoduodenectomy (PD). Of these, four were L-shaped PPPV with a thin wall that was difficult to isolate, while only one normal-shaped PPPV was reported previously. For patients undergoing PD, recognizing this variation is important to prevent PPPV injury, which could lead to liver ischemia or intraoperative hemorrhage. We here present a case of normal-shaped PPPV who underwent PD.

**Case presentation:**

A 68-year-old woman underwent PD for bile duct carcinoma at our hospital. Preoperative enhanced CT revealed that the portal vein was located anterior to the pancreas and posterior to the duodenum, and the L-shaped splenic vein was longitudinally located posterior to the pancreatic neck. During surgery, there was a loose tissue area between the PPPV and the pancreatic head, and the PPPV could be isolated safely. The morphology of PPPV was similar to normal portal vein. Due to the presence of the PPPV, a superior mesenteric artery (SMA)-first approach from the anterior was at high risk of vascular injury, and the pancreatic neck could not be dissected at the dorsal face of PV. Therefore, the SMA was revealed by the classic right posterior approach after transection of the pancreatic neck on the dorsal surface of L-shaped spleen vein, and the specimen was successfully resected without significant intraoperative bleeding. The patient was discharged 18 days after surgery without complications. The final pathology was bile duct carcinoma with R0 resection.

**Conclusion:**

PPPV is a rare variant that can be diagnosed by preoperative imaging. In PD procedure, knowledge of PPPV helps in surgical decision-making, approach selection and avoid major bleeding due to PPPV injury. The origin of normal-shaped and L-shaped PPPV might be different. Normal-shaped PPPV can be safely isolated in this case.

## Introduction

Prepancreatic postduodenal portal vein (PPPV) is a rare congenital variant of portal vein system, with only 16 cases reported in the literature and five of them undergoing pancreaticoduodenectomy (PD) (1). Of these, four were L-shaped PPPV with a thin wall that was difficult to isolate, while only one normal-shaped PPPV was reported previously (2). Recognition of PPPV is critical in surgical planning, approach selection and avoiding vascular injury. We here report the second normal-shaped PPPV that could be isolated safely during PD procedure, in accordance with the CARE Guidelines (3).

## Case presentation

A 68-year-old Chinese female was admitted to our hospital with abdominal pain and jaundice as the chief complaint. Her medical history included a hysterectomy for uterine fibroids 20 years ago, a history of hypertension with well-controlled blood pressure, left ventricular dilatation with complete left bundle branch block detected 2 years ago, and good physical activity. No other significant findings were noted in terms of family history, psychosocial history, and lifestyle history. On physical examination, the vital signs were normal, the skin and sclera were yellowish, no mass was palpable in the upper abdomen, no tenderness was found in the abdomen, and Murphy’s sign was negative. Laboratory tests revealed elevated total bilirubin (136.1 umol/L), carbohydrate antigen (CA) 19-9 (210.62 IU/mL), and N-terminal pro-B-type natriuretic peptide (NT-ProBNP) levels (3,067 pg./mL).

**Figure 1:**
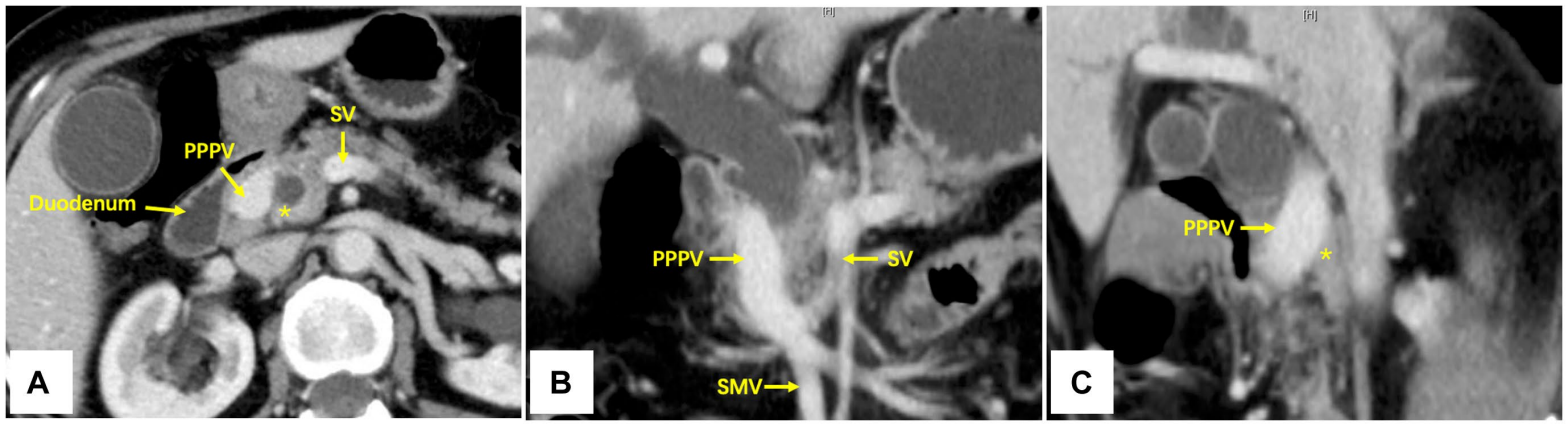
Axial **(A)**, coronal **(B)**, and sagittal **(C)** imaging of contrast-enhanced CT scan. The PPPV was located anterior to the pancreas and posterior to the duodenum, and the L-shaped SV was longitudinally located posterior to the pancreatic neck. PPPV, prepancreatic postduodenal portal vein; SV, spleen vein; SMV, superior mesenteric vein; *The pancreas posterior to PPPV.

**Figure 2:**
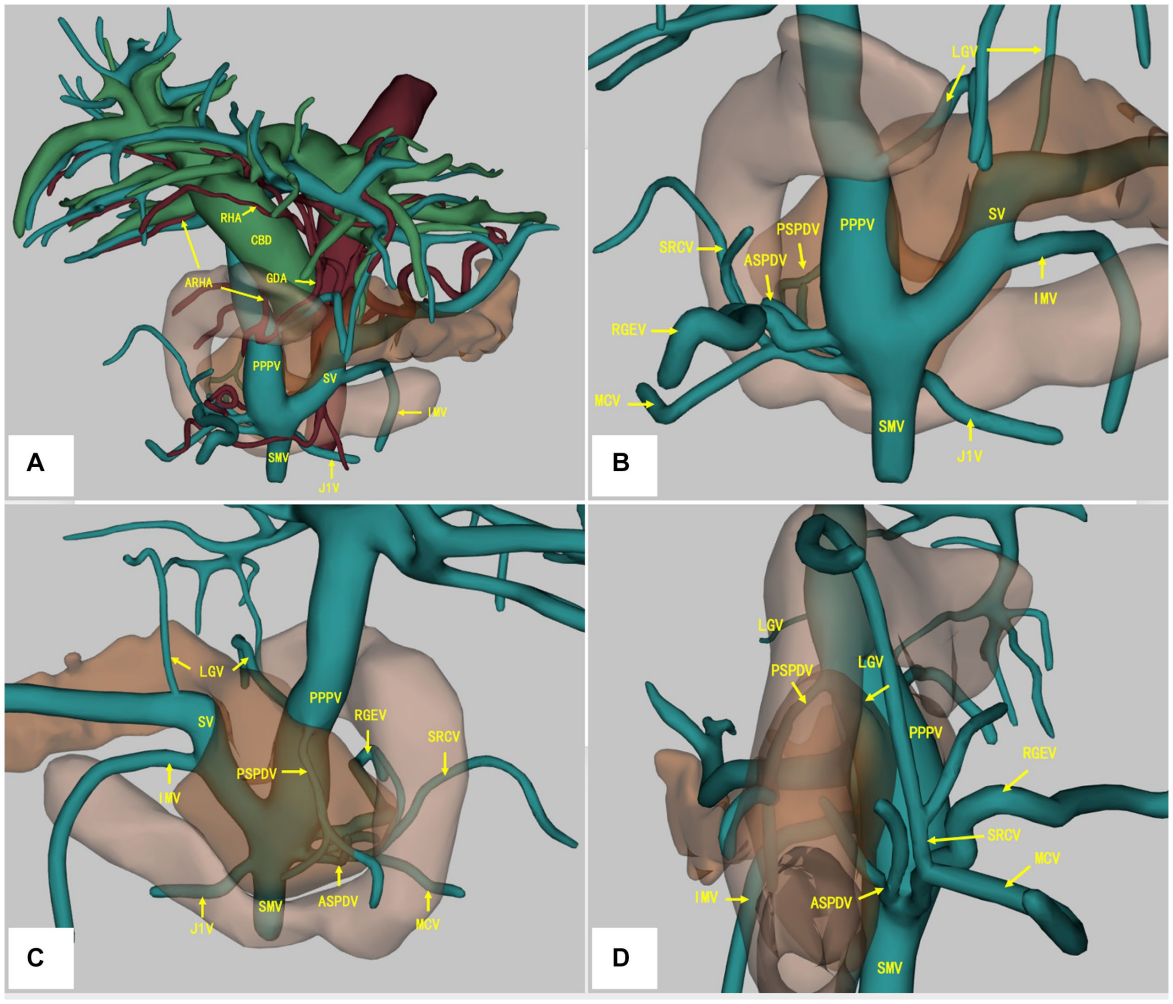
Three-dimensional vascular reconstruction of contrast-enhanced CT scan. The PPPV was located anterior to the pancreas and posterior to the duodenum, and the L-shaped SV was longitudinally located posterior to the pancreatic neck **(A)**. The RHA was located anterior to the CBD, and the ARHA originated from GDA was located posterior to the PV **(A)**. The branches of PV/SMV system were depicted from the anterior **(B)**, posterior **(C)**, and right side **(D)**. PPPV, prepancreatic postduodenal portal vein; SV, spleen vein; SMV, superior mesenteric vein; LGV, left gastric artery; PSPDV, posterior superior pancreaticoduodenal vein; ASPDV, anterior pancreaticoduodenal superior pancreaticoduodenal vein; SRCV, superior right colonic vein; RGEV, right gastroepiploic vein; MCV, middle colon vein; J1V, the first jejunal vein; RHA, right hepatic artery; ARHA, accessory right hepatic artery; GDA, gastroduodenal artery; CBD, common bile duct.

A contrast-enhanced CT scan of the abdomen revealed a tumor located at the lower bile duct with dilated intrahepatic bile ducts (malignancy suspicious). The portal-phase imaging showed that the portal vein (PV) was located anterior to the pancreatic head and posterior to the duodenum, and the L-shaped splenic vein (SV) was longitudinally located posterior to the pancreatic neck (Figures 1A–C), with no vascular invasion of the tumor. Three-dimensional vascular reconstruction revealed that the PPPV was located anterior to the head of the pancreas, posterior to the duodenum, and on the right side of the common bile duct (Figure 2A).

Moreover, an accessory right hepatic artery (ARHA) originated from gastroduodenal artery (GDA) was found to be located posterior to the portal vein, whereas the right hepatic artery (RHA) was located anterior to the common bile duct (CBD) and PPPV (Figure 2A). More detailed vascular anatomy of the PV system was depicted in the three-dimensional vascular reconstruction, including the left gastric artery (LGV), posterior superior pancreaticoduodenal vein (PSPDV), anterior pancreaticoduodenal superior pancreaticoduodenal vein (ASPDV), superior right colonic vein (SRCV), right gastroepiploic vein (RGEV), middle colon vein (MCV), the first jejunal vein (J1V) (Figures 2A–D). No IPDV was found in the CT scan.

The echocardiogram showed an enlarged left heart with diffusely reduced systolic amplitude and a left ventricular ejection fraction (LVEF) of 24%. A 24 h dynamic electrocardiogram (Holter) revealed a sinus rhythm with occasional premature polymorphic ventricular beats and complete left bundle branch. After cardiologist consultation, coronary angiography was performed without obvious coronary stenosis and the LVEF was 36% as suggested by LV angiography. Following multidisciplinary discussion including cardiologist and anesthesiologist, it was concluded that there was no absolute contraindication to surgery. Consequently, the preoperative diagnosis was common bile duct cancer, obstructive jaundice, intrahepatic bile ducts dilatation, post-hysterectomy, hypertension and chronic cardiac insufficiency.

**Figure 3:**
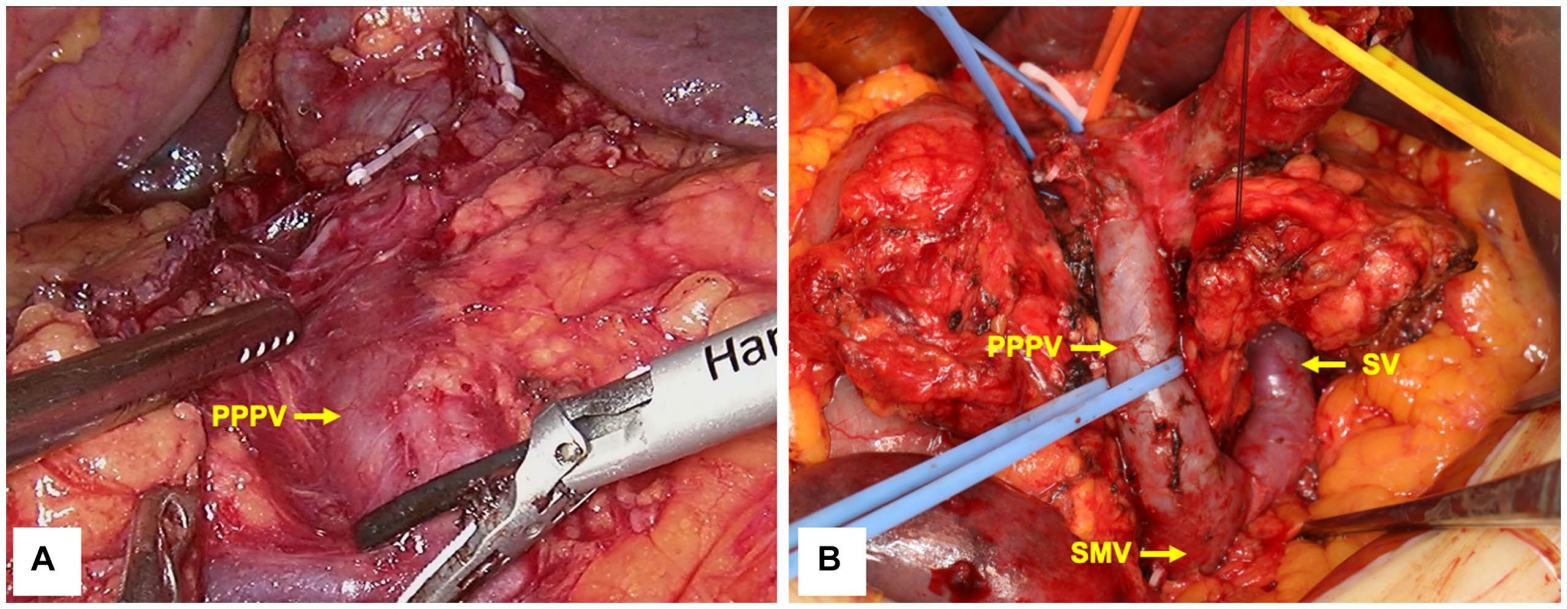
Intraoperative imaging before **(A)** and after **(B)** PPPV isolation. PPPV was located anterior to the pancreatic head, whereas SV was located posterior to the pancreatic neck. PPPV, prepancreatic postduodenal portal vein; SV, spleen vein; SMV, superior mesenteric vein.

A laparoscopic pylorus-preserving pancreaticoduodenectomy (PPPD) was performed as planned. After dissection of the duodenum, the PPPV was found to be located anterior to the pancreatic head, posterior to the duodenum, and on the right side of the common bile duct (Figure 3A), which was consistent with preoperative imaging. Due to the high risk of laparoscopic isolation of the PPPV from the pancreatic head, an open surgery (10 cm incision in the middle of the epigastrium) was performed to avoid vascular injury. We found that there was a loose tissue gap between the PPPV and the dorsal side of the pancreatic head, and the thickness of the PPPV vessel wall was normal. Consequently, we isolated the PPPV safely with no vascular injury (Figure 3B). The ARHA was unable to be preserved because the GDA had to be dissected and ligated to perform PPPD. After the dissection of GDA, there was no sign of ischemia of the right liver. Because of the anatomical variation, a superior mesenteric artery (SMA)-first approach from the anterior was at high risk, and the site to dissect the pancreatic neck was difficult to determine. The L-shaped SV was found to be located longitudinally posterior to the pancreatic neck (where the normal PV should be), so we established the post-pancreatic tunnel dorsal to the splenic vein to dissect the pancreatic neck. After dissecting the pancreatic neck, a traditional right posterior approach (through Kocher’s incision) was performed to reveal the SMA and resect the uncinate process. The pancreas was soft with a main pancreatic duct diameter of 1 mm. A double-layer duct-to-mucosa pancreatojejunostomy, a cholangiojejunostomy, and a duodenojejunostomy were performed, respectively. The operative time was 379 min, with an estimated intraoperative blood loss of 400 mL.

The patient experienced no postoperative complications, including pancreatic fistula. The amylase level of the drainage fluid was normal on postoperative day (POD) 3 and no postoperative pancreatic fistula was observed. The abdominal drain was removed at POD 12 and the patient was discharged at POD 18. The postoperative pathology was confirmed as intermediate differentiated cholangiocarcinoma with R0 margin. At 12 months postoperative follow-up, the patient survived without tumor recurrence.

## Discussion

PPPV is a rare variant of the portal vein system, and this is the 17th case reported worldwide and the sixth case of PPPV undergoing PD. Unlike the previously reported L-shaped PPPV, the present case has a normal-shaped PPPV which could be isolated safely. To our best knowledge, this is the second case of a normal-shaped PPPV undergoing PD with successful isolation of the PPPV.

The embryonic development of the portal system was described by Marks (4). At 4–6 weeks, the venous blood from the foregut is drained by a pair of parallel vitelline veins which are connected by three anastomoses: the cranial anastomosis intrahepatically, the middle anastomosis behind the duodenum, and the caudal anastomosis in front of the duodenum. Normally, both the ventral and dorsal pancreatic buds are located ventrally to the vitelline veins. With the clockwise rotation of the ventral pancreatic bud to fuse with the dorsal pancreatic buds (viewed from a cephalad perspective), the cephalic part of the right vitelline vein, the caudal part of the left vitelline vein and the caudal anastomosis disappear, leaving the caudal part of the vitelline vein, the middle anastomosis and the cephalic part of the right vitelline veins to form the PV behind the pancreas. In PPPV cases, the dorsal pancreatic bud is initially positioned dorsally to the left vitelline vein, and subsequently the PV is located anterior to the pancreas and posterior to the duodenum after the rotation of the dorsal pancreatic bud (5).

PPPV was first reported by Brook and Gardner (6), and a total of 17 cases have been reported to date, including the present case. We have summarized all cases to date and our cases in Table 1 (1, 2, 5–17). Among them, 11 were reported as L-shaped PPPV, while only three were normal-shaped PPPV. Of the 11 L-shaped PPPV cases, three were co-existent with the presence of a normal PV (posterior to the pancreatic neck) which enter into several hepatic segments. A total of 12 patients underwent surgery, of which six underwent PD. Among them, four had the carcinoma of the bile duct and two had the carcinoma of ampulla of Vater. In terms of PPPV morphology, four cases were reported as L-shaped (1, 5, 8, 15) and two cases were normal-shaped (2) (including the present case). All four cases of L-shaped PPPV had a much thinner venous wall, with close adhesion to pancreas and difficult to isolate. Therefore, three of them underwent PPPV resection and reconstruction after attempting to isolate the PPPV due to intraoperative massive hemorrhage (1, 8, 15), and one case underwent R2 resection with PPPV preserved (5). In the present case and Tanaka’s report (2), the normal-shaped PPPV had normal thickness of venous wall and there was a loose tissue area posterior to PPPV and anterior to the pancreatic head which could be isolated safely. Therefore, we propose the hypothesis that the L-shaped PPPV and normal-shaped PPPV may have different origins, since they have different vessel wall thickness and morphology, and should be considered as two different portal vein variants. In the case of L-shaped PPPV, isolation would cause fatal hemorrhage and PPPV should be resected and reconstructed, even in the absence of tumor invasion. In contrast, for normal-shaped PPPV, isolation can be performed safely without the need for PPPV resection and reconstruction to avoid postoperative complications such as PV thrombosis.

**Table 1:** Seventeen cases of PPPV.

References	Year	Age	Gender	Diagnosis	PPPV shape	Co-existence of normal PV	Surgery	PPPV isolation
Brook and Gardner (6)	1972	84	F	Choledocholithiasis	–	No	Cholecystectomy	–
Matsumoto et al. (5)	1983	64	M	Carcinoma of the bile duct	L-shaped	No	PD	Not attempted
Dumeige and Farret (7)	1989	49	M	Chronic pancreatitis	–	–	laparotomy	–
Matsui et al. (8)	1995	66	F	Carcinoma of the bile duct	L-shaped	Yes	PD + PV resection	Fail
Yasui et al. (9)	1998	65	M	Cecal cancer	L-shaped	No	Colon resection	–
Ozeki et al. (10)	1999	62	F	Liver metastasis from rectal cancer	L-shaped	No	Hepatectomy	–
Tanaka et al. (2)	2000	51	M	Carcinoma of the bile duct	Normal-shaped	No	PD	Success
Inoue et al. (11)	2003	50	M	Gastric cancer	L-shaped	No	Gastrectomy	–
Jung et al. (12)	2005	28	F	Cholelithiasis	L-shaped	No	Cholecystectomy	–
Tomizawa et al. (13)	2010	74	M	Colorectal metastasis to the liver	Normal-shaped	No	None	–
Tomizawa et al. (13)	2010	74	F	Breast cancer	L-shaped	No	None	–
Jain et al. (14)	2013	56	F	Autoimmune hepatitis	L-shaped	No	None	–
Shimizu et al. (15)	2014	85	F	Carcinoma of ampulla of Vater	L-shaped	No	PD + PV resection	Fail
Goussous and Cunningham (16)	2017	55	F	Choledocholithiasis	–	No	ERCP	–
Higashihara et al. (1)	2022	73	M	Carcinoma of ampulla of Vater	L-shaped	Yes	PD + PV resection	Fail
Kitagawa (17)	2022	40	M	Normal	L-shaped	Yes	None	
Current case	2023	68	F	Carcinoma of the bile duct	Normal-shaped	No	PD	Success

PPPV, prepancreatic postduodenal portal vein; PV, portal vein; PD, pancreaticoduodenectomy; ERCP, endoscopic retrograde cholangio-pancreatography.

In addition, the existence of PPPV may affect the selection of surgical approach and the site of pancreatic neck dissection. The SMA-first approach should be avoided due to the altered anatomy anterior to the SMA and the unclear relative position to prevent vascular injury. Additionally, the post-pancreatic tunnel on the surface of PV cannot be established due to the absence of PV posterior to the pancreatic neck. In this case, the L-shaped SV was longitudinally located posterior to the pancreatic neck (where the normal PV should be), so the post-pancreatic tunnel was established on the dorsal surface of splenic vein to dissect the pancreatic neck. The SMA was revealed by a classic right posterior approach (through Kocher’s incision), which is not affected by the variation of the portal vein system, thus minimizing the risk of variant vessel injury and ensuring patient safety.

This study has several limitations. Firstly, due to the rarity of reported cases of PPPV, the distinction between L-shaped and normal-shaped PPPV needs to be further confirmed. Secondly, the distinction between the origins of L-shaped and normal-shaped PPPV was not discussed in this study. Finally, the mechanism for the coexistence of normal PV in some reports is unclear and not discussed in the current study. The value of this study is the report of the second case of normal-shaped PPPV undergoing PD and the suggestion that normal-shaped PPPV can be safely isolated.

In conclusion, PPPV is a rare portal vein anomaly that can be easily diagnosed by preoperative imaging. Awareness of PPPV may prevent injury or dissection of this variant vessel, which could result in fatal hemorrhage and liver ischemia. Preoperative diagnosis of PPPV is essential for surgical planning, including the selection of approach and site of dissection of the pancreatic neck, and the choice of resection/reconstruction or isolation depending on the PPPV morphology. Our experience suggests that normal-shaped PPPV can be safely isolated.

## Data availability statement

The original contributions presented in the study are included in the article/supplementary material, further inquiries can be directed to the corresponding author.

## Ethics statement

Written informed consent was obtained from the individual(s) for the publication of any potentially identifiable images or data included in this article.

## Author contributions

BT and SL wrote the first draft of the manuscript. PW, JS, and XW contributed to conception and design of the study. XW revised the manuscript. JM collected the data. All authors contributed to the article and approved the submitted version.

## Funding

This work was supported by the grants from the Chinese Academy of Medical Sciences Innovation Fund for Medical Sciences [grant number: 2019-I2M-5-056] and National Natural Science Foundation of China [grant numbers: 81930119, 82090050, and 82090053].

## Conflict of interest

The authors declare that the research was conducted in the absence of any commercial or financial relationships that could be construed as a potential conflict of interest.

## Publisher’s note

All claims expressed in this article are solely those of the authors and do not necessarily represent those of their affiliated organizations, or those of the publisher, the editors and the reviewers. Any product that may be evaluated in this article, or claim that may be made by its manufacturer, is not guaranteed or endorsed by the publisher.
